# Radiomics for the Detection of Active Sacroiliitis Using MR Imaging

**DOI:** 10.3390/diagnostics13152587

**Published:** 2023-08-03

**Authors:** Matthaios Triantafyllou, Michail E. Klontzas, Emmanouil Koltsakis, Vasiliki Papakosta, Konstantinos Spanakis, Apostolos H. Karantanas

**Affiliations:** 1 Department of Medical Imaging, University Hospital of Heraklion, 71110 Heraklion, Greece; manthostr@gmail.com (M.T.); miklontzas@gmail.com (M.E.K.); emmanouilkolts@gmail.com (E.K.); vasopapacosta@gmail.com (V.P.); vispan@windowslive.com (K.S.); 2 Department of Radiology, School of Medicine, University of Crete, Voutes Campus, 71500 Heraklion, Greece; 3 Department of Radiology, Karolinska University Hospital, 17164 Stockholm, Sweden

**Keywords:** active sacroiliitis, axial spondyloarthropathy, radiomics, machine learning, bone marrow edema

## Abstract

Detecting active inflammatory sacroiliitis at an early stage is vital for prescribing medications that can modulate disease progression and significantly delay or prevent debilitating forms of axial spondyloarthropathy. Conventional radiography and computed tomography offer limited sensitivity in detecting acute inflammatory findings as these methods primarily identify chronic structural lesions. Conversely, Magnetic Resonance Imaging (MRI) is the preferred technique for detecting bone marrow edema, although it is a complex process requiring extensive expertise. Additionally, ascertaining the origin of lesions can be challenging, even for experienced medical professionals. Machine learning (ML) has showcased its proficiency in various fields by uncovering patterns that are not easily perceived from multi-dimensional datasets derived from medical imaging. The aim of this study is to develop a radiomic signature to aid clinicians in diagnosing active sacroiliitis. A total of 354 sacroiliac joints were segmented from axial fluid-sensitive MRI images, and their radiomic features were extracted. After selecting the most informative features, a number of ML algorithms were utilized to identify the optimal method for detecting active sacroiliitis, leading to the selection of an Extreme Gradient Boosting (XGBoost) model that accomplished an Area Under the Receiver-Operating Characteristic curve (AUC-ROC) of 0.71, thus further showcasing the potential of radiomics in the field.

## 1. Introduction

Diagnosing sacroiliitis involves a combination of clinical and imaging findings, often requiring collaboration between specialists, as demonstrated by the Assessment of SpondyloArthritis International Society (ASAS) criteria [1]. Conventional radiography (CR) was the first modality utilized for axial spondyloarthritis (axSpA) detection [2]. However, it is confined to findings like subarticular sclerosis or erosions, joint space narrowing, and ankylosis, which demonstrate at an advanced stage of the disease [3]. This is also the case with computed tomography (CT), which offers more accurate and prompt detection of chronic structural osseous lesions but exposes patients to more radiation and fails to detect active sacroiliitis.

The principal symptoms and signs of active inflammation include enthesitis and pain in an affected area. Those two characteristics are under no circumstances specific to a particular disease and can be attributed to other processes like trauma or wear and tear strain. The list of potential diagnoses includes osteoarthritis, insufficiency fractures, and neoplastic and infectious processes, all of which require radically different therapeutic interventions. Hence, the diagnosis of sacroiliitis is a combination of clinical and imaging findings, as they are stated in the ASAS criteria, and often requires the cooperation of specialists [1].

Magnetic Resonance Imaging (MRI) is the preferred modality for diagnosing axSpA. It is excellent in demonstrating inflammatory lesions such as synovitis, enthesitis, and structural lesions, including sclerosis (hypointense lesions extended greater than 5 mm from a joint), erosions, periarticular fat deposition, and ankylosis, which are indicative of chronic inflammation and degeneration. The main role of MRI in the diagnostic criteria of ASAS is to support the diagnosis of active sacroiliitis when bone marrow edema (BME) or osteitis is identified [4,5] in the bone adjacent to the sacroiliac joints [1,2,3,4,5,6]. Nonetheless, differentiation between BME related to axSpA and BME related to other conditions, such as joint degeneration, requires significant expertise in musculoskeletal imaging and a combination of clinical and imaging features of the disease [7]. Timely diagnosis of inflammatory sacroiliitis permits timely prescription of disease-modulating drugs and biologic disease-modifying antirheumatic drugs, significantly improving outcomes [8]. Meanwhile, degenerative sacroiliitis can be managed with pain relief and physical therapy, or, in some cases, with minimally invasive techniques [9].

Radiomics, which are high-dimensional quantitative features derived from medical images, can be utilized in diagnostic, predictive, and prognostic models, predominantly in oncology [10]. They allow high-fidelity analysis of regions of interest on medical images with the potential to offer image-based biopsy of target lesions. This study explores the possibility of using radiomics-based models to detect inflammatory sacroiliitis in MR images and differentiate inflammatory BME from other causes. The development of such an algorithm would provide a valuable tool for radiologists, particularly those not explicitly trained in this area.

## 2. Materials and Methods

### 2.1. Dataset

**Figure 1:**
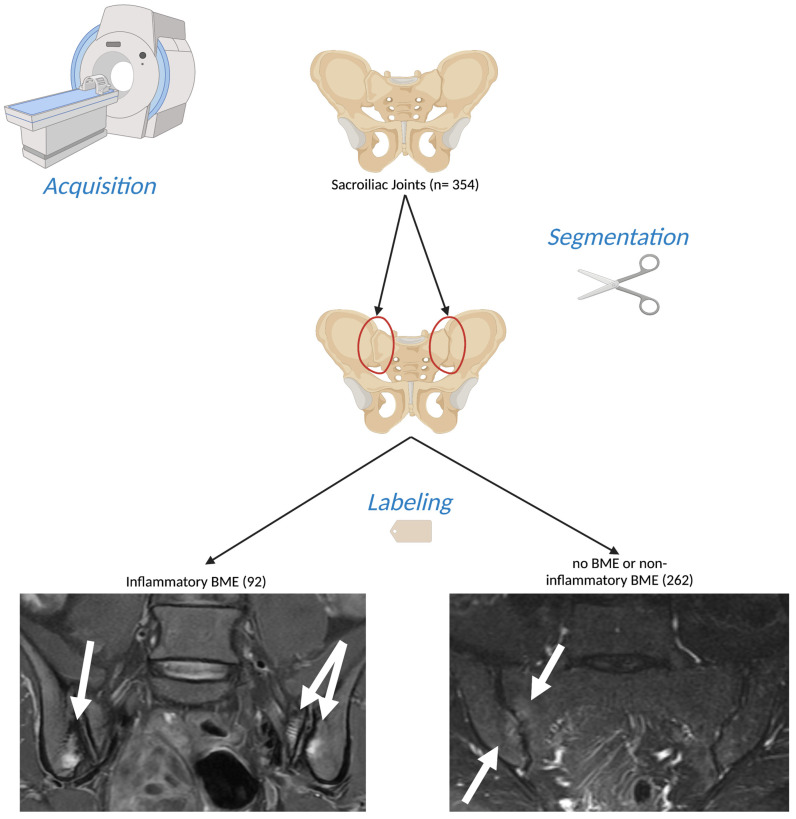
Illustration of the process of data acquisition and preparation in this study. Oblique coronal (left) and oblique axial (right) Short Tau Inversion Recovery (STIR) Magnetic Resonance (MR) images from patients scanned between January 2017 and September 2021 were retrospectively collected. Each sacroiliac joint was individually segmented, resulting in 354 segments. Among these, 92 segments were classified as positive for inflammatory bone marrow edema (BME), while the remaining segments were marked as negative. (Created with BioRender [11], 17 June 2023).

A dataset composed of oblique axial Short Tau Inversion Recovery (STIR) and Proton Density Fat Saturated (PD-FS) measurements of *n* = 177 individuals was used (Figure 1). The images were acquired using a 1.5 T MR scanner (Vision/Sonata, Siemens, Erlangen, Germany) between January 2017 and September 2021. They were subsequently retrieved from the PACS of the University Hospital of Heraklion in September 2021 in a retrospective manner. The exclusion criteria included tumors extending to the sacroiliac joints, septic sacroiliitis, previous radiotherapy, and cases with traumatic or insufficiency pelvic fractures. All other sacroiliac joint examinations from our database were included in the study. It is important to note that this study adhered to the principles outlined in the Declaration of Helsinki and received institutional review board approval; informed consent was waived due to the retrospective anonymized nature of the study. Interestingly, no scan was excluded based on image quality or the presence of artifacts. This approach was taken to ensure that the algorithm we developed was trained and tested under conditions that mirrored real-world practice as closely as possible.

### 2.2. Imaging Section

The sacroiliac joints of each patient were evaluated by two highly experienced musculoskeletal radiologists: AHK, who has accumulated 40 years of experience, and KS, with 10 years of experience in the field. They classified each joint as either exhibiting inflammatory BME, indicative of spondyloarthritis (SpA), or as negative—either due to the absence of BME or the presence of BME resulting from non-inflammatory causes. The evaluations from these experienced professionals are considered the gold standard for diagnosing sacroiliitis.

These expert evaluations were based on the European Alliance of Associations for Rheumatology (EULAR) criteria [8]. A sacroiliac joint was classified as showing signs of inflammation related to SpA if BME was observed on a minimum of two adjacent slices, or alternatively, if it was visible in at least two distinct locations within a single slice. The presence of BME is especially notable if located within the areas of the sacroiliac joint commonly affected by SpA, specifically the subchondral or capsular regions of the joint, primarily at the lower levels. Moreover, it is noteworthy that BME is frequently seen in conjunction with other MRI manifestations of sacroiliitis in SpA patients, such as enthesitis in the pelvis and lower spine and structural abnormalities like erosions, subarticular sclerosis, fatty metaplasia, and ankylosis.

It is also crucial to note that AHK and KS were blind to the clinical assessments made by the prescribing physicians when conducting the evaluations, ensuring the radiologists’ judgments were unbiased and solely based on their expert interpretation of the MRI scans.

### 2.3. Segmentation and Feature Extraction

Radiomics data were derived from regions of interest (RoIs) that were manually designated by three radiology residents (EK, VP, and MT) using the 3D Slicer software (version 4.11 for Windows, with the last access date being 20 December 2021) [12]. Prior to this, all operators received training in the segmentation process from an individual experienced in medical imaging research, with over a decade of experience in the field (MEK) And radiomics were extracted using the Radiomics extension of 3D Slicer [13], employing a voxel size of 1 × 1 × 1 mm for the entire dataset. A bin width of 64 was chosen to strike a balance between preserving valuable information and minimizing excessive noise. The resulting dataset comprises 940 features, encompassing a range of original, wavelet, and Laplacian of Gaussian-filtered features. These features vary from easily interpretable first-order features to more complex higher-order texture features, as suggested by the current literature [14]. 

The extracted data were standardized to mitigate any potential distortion in model training and reported accuracy caused by numerical value differences. Standardization ensures that data are transformed to have a mean of zero and a standard deviation of one, allowing for fair and unbiased comparison between features [15]. This step enhances the reliability and interpretability of the results, enabling a more accurate assessment of the predictive performance. 

**Figure 2:**
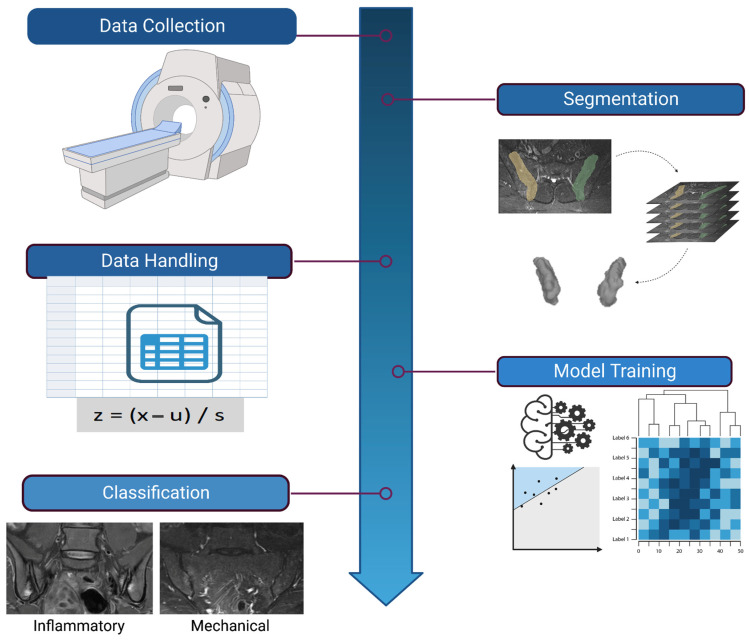
Overview of the complete pipeline employed in this study. The process begins with image acquisition and retrospective data collection. Segmentation is performed next by individually processing each sacroiliac joint. Feature extraction follows, allowing us to retrieve a wide array of features from each segment. The data are then preprocessed, including a standardization step to normalize the range of feature values. The feature selection phase ensues, including using Pearson correlation and then the Boruta method to pinpoint the most significant features. The pipeline culminates in model training, where machine learning (ML) algorithms are trained on the selected features and used to classify each segment as either positive or negative for inflammatory BME (Created with BioRender [11], 18 June 2023).

Notably, our dataset displays a significantly higher count of negative cases compared to positive ones, a pattern also seen in the literature [16], as well as other radiomics studies [17]. This discrepancy led to concerns about potential biases during algorithm training. To address this, we employed a stratified sampling strategy, thus ensuring that both the training and test sets exhibited similar distributions of positive and negative cases [18]. The final split was performed using an 80:20 ratio for training to testing data. An overview of the pipeline followed in this study is given in Figure 2.

### 2.4. Feature Selection

To identify salient features, we incorporated a variety of feature selection methodologies. The process kicked off with Pearson correlation, a filter feature selection method. Features that were weakly correlated with the target variable, according to a predetermined threshold of 0.8, were eliminated. This preliminary process reduced the feature count from an initial 940 to 205, preserving those with a significant relationship with the target variable. The feature selection process continued with the Boruta method with the *p*-value threshold set at 0.01, thereby delivering the most favorable results according to the trained models’ metrics. Stemming from the Random Forest algorithm, Boruta is well regarded for its adaptability and impressive outcomes across diverse problem domains [19]. It operates by modifying the original dataset, creating “shadow features”. Only shadow features surpassing a certain importance threshold are selected for further analysis [20]. Boruta directly selects features from the original feature space, thus improving interpretability and fostering trust, both of which are vital in the medical domain.

### 2.5. Machine Learning

A Jupyter notebook served as our main tool for managing and processing the dataset, which involved tasks such as standardization and subsequent manipulations like dimensionality reduction and model training. We utilized Python programming language v3.11.3 for these tasks [21]. To mitigate the risk of “snooping”—the inadvertent leakage of information from the test set that might lead to a form of overfitting—we partitioned our data into training and test sets (with a ratio of 80/20) prior to standardization. The train–test split is necessary to ensure that a model is tested on a different set than the one it is trained on, and, hence, the results are not the outcome of learning the training set’s noise. 

Support Vector Machine (SVM) and Logistic Regression were utilized, as well as a Random Forest (RF) architecture, and finally, we applied a gradient boosting method via the robust Extreme Gradient Boosting (XGBoost) algorithm.

Hyperparameter optimization was performed with Random Search, a stochastic, non-exhaustive tuning method. This approach examines a subset of all possible combinations, thus preserving crucial memory and computational resources, while still maintaining high-quality results.

Logistic Regression is a frequently employed machine learning method used to model the likelihood of a binary outcome. It ascertains the probability of an event by fitting data to a logistic function. Essentially, it serves to comprehend the relationship between the input features and the binary output variable, such as a positive or a negative outcome for inflammatory bone marrow edema (BME). SVM has proven its efficacy in research on the same clinical question [22]. It determines the optimal hyperplane for efficient class separation by maximizing the distance of the classes from the selected hyperplane. The lines that pass through the closest observations to the chosen hyperplane are known as the support vectors. 

RFs, consisting of a set of non-correlated decision trees, are applicable for both regression and classification tasks. Their interpretability makes them a favorable choice for various radiomics applications using tomographic methods [23,24]. RFs work by building numerous decision trees during training and outputting the mode of the classes (classification) or the mean prediction (regression) of individual trees. The inherent randomness of their design, coupled with their ensemble nature, enhances the model’s ability to generalize, thereby mitigating overfitting risks. In our study, the proposed RF-based model outperformed both Logistic Regression and SVM.

Gradient boost models construct decision trees of a predetermined size, which evolve based on the errors of the preceding trees. In classification tasks, the initial prediction is the log of the odds. After subtracting observed values from the initial prediction, the model generates residuals that represent prediction errors. New decision trees are then trained to measure a new set of residuals. The residuals of subsequent trees shrink, implying that the error is progressively being minimized as the model approaches the desired outcome. An essential hyperparameter of gradient boosting is the learning rate, which guarantees smaller yet more controlled refinement by scaling each tree’s contribution to the training process. The combination of the aforementioned trees, as guided by the preset learning rate, results in a high-performing ensemble machine learning model. In similar classification tasks, XGBoost algorithms have proven their robustness [25,26] by outperforming other methods.

### 2.6. Statistical Analysis

Receiver Operating Characteristic (ROC) curves were constructed within a Jupyter Notebook environment, utilizing the sklearn.metrics module from the Python Scikit-learn package. The performance of the various classification models was evaluated based on the Area Under the Curve (AUC) of these ROC curves. This approach provided a robust measure of model performance, allowing us to effectively compare the different models. Visualization of these ROC curves was achieved with the assistance of the Matplotlib library. Sensitivity, specificity, positive predictive value (PPV), negative predictive value (NPV), accuracy (ACC), and F1 score were calculated for the model with the highest AUC-ROC score, with the use of Numpy and Matplotlib libraries [27,28]. Ninety-five percent confidence intervals were calculated for each AUC using a bootstrapping method with 1000 iterations. This process, analogous to generating new datasets from an original dataset through random sampling with replacement, was implemented to validate the stability of our model and its prediction accuracy.

## 3. Results

The MR images of 354 sacroiliac joints of 177 individuals were included in our study. Radiomics feature extraction was performed utilizing the aforementioned MR images.

### 3.1. Selected Features

**Figure 3:**
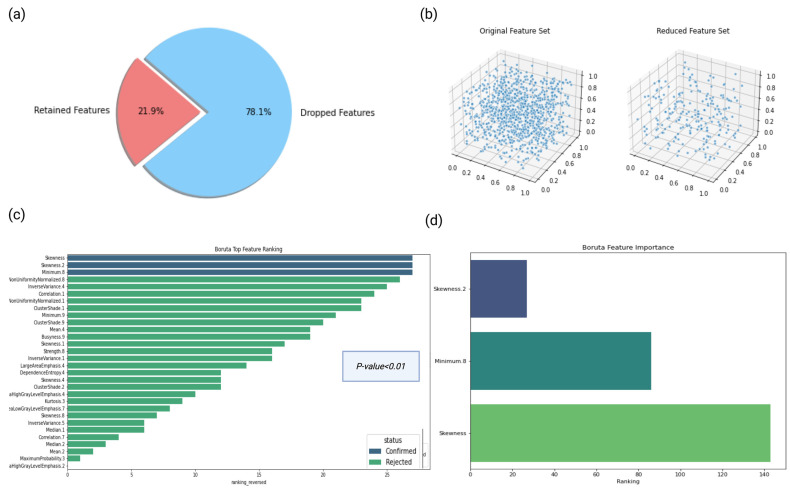
Feature selection process used in this study. The process starts with 940 features. Using Pearson correlation, features that are highly correlated are eliminated, reducing the count to 205, as shown in the charts (**a**,**b**). Subsequently, the Boruta method is applied (**c**), which delivers the top 3 representative features for further analysis (**d**). (Created with BioRender [11], 18 June 2023).

**Figure 4:**
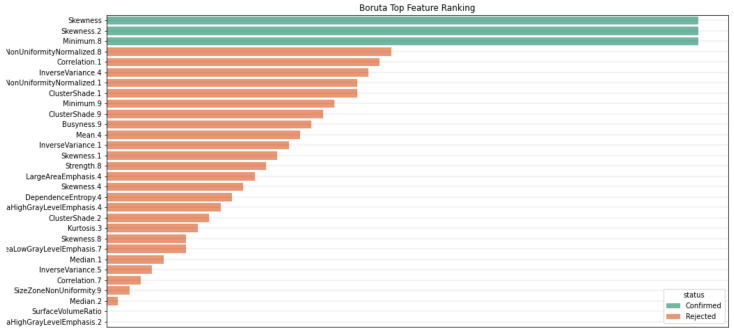
Features identified as important by Boruta with a *p*-value threshold of 0.01. Two of these features are related to skewness, a measure capturing the asymmetry in pixel intensity distribution in MR images. The algorithm, thus, aids the model in distinguishing between normal tissues and those affected by edema.

Through this procedure, we identified the top three representative features, two of which were related to skewness (Figure 3 and Figure 4). The final features were subsequently incorporated into various classification models. The three features identified as being more important using Boruta included skewness, a wavelet transformation of skewness (skewness 2) and a wavelet transformation of the first-order minimum feature. Skewness assesses how asymmetrically the gray values are distributed around the mean, while the minimum feature represents the minimum gray value in a voxel (Figure 3).

### 3.2. Model Results

**Figure 5:**
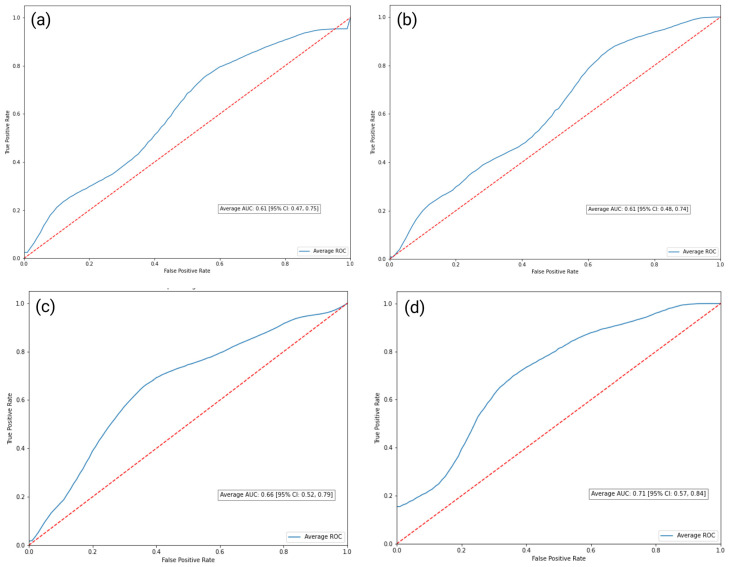
Schematic representation of the classification methods employed for the differentiation of inflammatory BME. Initially, simple linear models like Logistic Regression (**a**) and Support Vector Machine (SVM) (**b**) were selected, followed by Random Forest (RF) architecture (**c**), and, finally, culminating in the application of a gradient boosting method using the robust XGBoost algorithm (**d**). Each model’s performance was optimized by tuning its hyperparameters through a Random Search approach, which maintains high-quality results while conserving computational resources. The figure illustrates the relative performance of each method (SVM, Logistic Regression, RF, and XGBoost) using, as a performance metric, the Area Under the Receiver-Operating Characteristic curve (AUC-ROC), demonstrating the superior performance of the XGBoost model with an AUC-ROC of 0.71. (Created with BioRender [11], 18 June 2023).

The AUC-ROC scores are presented in the table below (Table 1). The Logistic Regression (Log Reg) and SVM models both scored an AUC of 0.61, with the 95% confidence intervals (CI) ranging between 0.47 and 0.75 and between 0.48 and 0.74, respectively. The proposed RF-based model achieved an AUC of 0.66, with a 95% CI ranging from 0.52 to 0.79. Finally, the XGBoost model accomplished an Area Under the ROC curve of 0.71, with a 95% CI ranging from 0.57 to 0.84 [29] (Figure 5).

**Figure 6:**
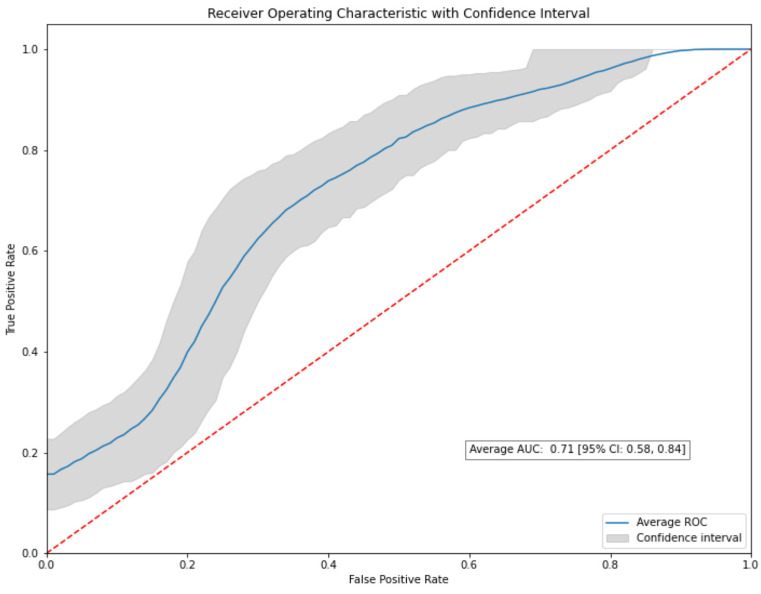
The ROC curve generated from the bootstrapped XGBoost model after 1000 resamples. The model consistently achieves an AUC of 0.71, reaffirming its performance from the non-bootstrapped data.

To further validate the outcomes of our study, we employed a bootstrapping method with a thousand resamples for all the models, with the number of iterations set to 1000. Remarkably, the XGBoost model sustained its performance through the bootstrapping process, consistently achieving an Area Under the ROC curve (AUC) of 0.71, mirroring the result from the non-bootstrapped data. Importantly, the bootstrapped model generated a 95% confidence interval (CI) of 0.58–0.84 for the AUC (Figure 6).

**Figure 7:**
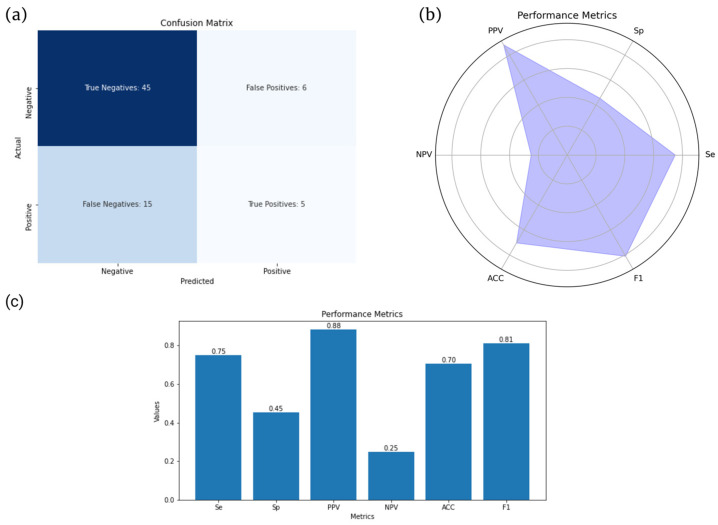
Comparison of key evaluation metrics for the XGBoost model. (**a**) Confusion matrix illustrating the performance of the model in classifying true positives, true negatives, false positives, and false negatives. (**c**) Bar plot displaying sensitivity (Se), specificity (Sp), positive predictive value (PPV), negative predictive value (NPV), accuracy (ACC), and F1 score. (**b**) Spider chart (or radar chart) visually representing the same metrics shown in (**b**).

Additional evaluation metrics for the XGBoost model are also provided below (Figure 7).

## 4. Discussion

In this study, radiomics was used as a method to identify inflammatory sacroiliitis. In our radiomics study, we observed varying performances across different classification models. The linear models, Logistic Regression and Support Vector Machine, yielded moderate-to-weak results in terms of their predictive power. In contrast, the Random Forest algorithm exhibited superior performance, outperforming both linear models. However, it was the XGBoost algorithm that emerged as the most effective. This algorithm reaffirmed its efficacy by surpassing all other classification models, demonstrating superior performance as evidenced by having the highest Area Under the Curve (AUC) among the classifiers tested. This underlines XGBoost’s potential as a robust tool for radiomics data classification in sacroiliitis studies. Importantly, our proposed model achieved a remarkable PPV of 88%, meaning that it would be quite useful as an ancillary tool to detect patients requiring disease-modulating treatment.

Inflammatory BME manifests as a high-intensity signal in fluid-sensitive MRI sequences due to fluid accumulation induced by inflammation within the bone marrow. Notably, it exhibits a more expansive spatial extent compared to edema caused by mechanical factors, which is in line with the European Alliance of Associations for Rheumatology (EULAR) criteria [30,31]. Skewness, a statistical measure of the asymmetry of gray value distribution, has already demonstrated its ability to detect intensity discrepancies and asymmetry in pixel intensities within regions of interest in T2-weighted images [32]. Within the setting of BME, we anticipate a positive skewness (or a rightward skew) due to the abundance of these high-intensity pixels. Consequently, the original skewness serves a crucial role in supporting the model’s ability to differentiate between healthy and edematous tissues by quantifying the distributional asymmetry of pixel intensities, a feature that is intrinsic to BME.

Our team is not the first one to study MR-based radiomics to diagnose active sacroiliitis (Table 2). Texture-derived radiomic features were used by Kepp et al. in 2021 to compare inflammatory and degenerative sacroiliitis. They further examined a third group consisting of healthy individuals, yielding promising results. Their study included a total of 90 patients. However, their most accurate outcomes were generated by a model that employed T1W-CE images [17]. This model which was built upon contrast-enhanced images demonstrated the highest accuracy, probably by detecting ancillary inflammatory lesions like synovitis or enthesitis, which could suggest inflammation [4]. An ongoing debate exists within the expert community regarding the necessity of including contrast-enhanced images for the specific clinical question under consideration [33,34,35]. We chose to develop a model that relies on fluid-sensitive images, as this approach would be more congruent with prevailing clinical practices. Similarly, Faleiros et al. utilized coronal STIR images from 56 patient scans to train their algorithm, and labeled these scans as either positive or negative for inflammatory sacroiliitis, depending on the presence of subchondral BME [22]. This study utilized a dataset comprising 43% of positive cases as opposed to approximately 25% in other studies. They employed ReliefF and Wrapper methods to identify the most representative features and tested several classifiers, including Support Vector Machines (SVM), k-Nearest Neighbors (k-NN), and Artificial Neural Networks (ANN). The most effective model turned out to be a Multilayer Perceptron (MLP) classifier, achieving an accuracy and a specificity rate of 0.8 and 0.667, respectively, in the validation sets. A similarly small dataset (47 cases) was also used by Tenorio et al. to distinguish between axial and peripheral aSpA [36], and they identified a feature, Tamura_D11_SD, as important in the diagnosis of axial aSpA and sacroiliitis. These retrospective results were based on a significantly smaller dataset, which bears an important chance of overfitting. 

Ye et al. also sought to establish a nomogram for the diagnosis of active sacroiliitis by integrating radiomics and clinical data [37]. However, nomograms come with several drawbacks. They are frequently employed in radiology research to handle complex tasks that exceed the capabilities of a simple linear model; however, they oversimplify these tasks and lack reliability since they demand numerous user-driven decisions [38]. In their study, Ye et al. provided their algorithm with approximately twice as many positive cases as negative ones, deviating considerably from the reported disease prevalence relative to mechanical causes [16]. This data imbalance could potentially introduce a bias in the model, skewing it toward a positive label. Ye et al. also excluded patients who had received treatment. In contrast, we opted to include such patients in our study. Our objective was to enable our model to detect acute BME of an inflammatory cause at any point during disease progression, as BME may resurface even after treatment. Another nomograph was introduced by Zheng et al. to quantify BME [39]. Their study included patients with axSpA, who were deemed positive according to the ASAS criteria [1]. Their goal was to develop a nomogram that could predict and categorize patients with SPARCC scores of either less than 2 or equal to and above 2 [40]. Their results indicate that their scoring provides superior metrics compared to the SPARCC system.

Previous work from our group utilized radiomics to differentiate among conditions related to bone marrow edema. Transient osteoporosis of the hip and avascular necrosis of the femoral head are conditions accompanied by varying degrees of bone marrow edema. We demonstrated that radiomics can differentiate between these two conditions with an accuracy that is the same or superior to radiologists [33]. An importance difference between the aforementioned conditions and sacroiliitis is that bone marrow edema in sacroiliitis is, in most cases, located on both sides of the sacroiliac joint, necessitating the segmentation of the subarticular bone on both sides of the joint. Another important difference is wavelet transformations of original features are important in transient osteoporosis and avascular necrosis because of the presence of fracture lines or band-like patterns [33]. In our case where no lines/edges exist in the areas of bone marrow edema, original features such as skewness are found to be important for the identification of inflammatory bone marrow edema.

Our radiomics study has strengths and limitations. The significant strengths of the study include a relatively large sample size and the use of non-enhanced MR images which are representative of routine radiological practice. The limitations include the retrospective nature of data collection that may lead to overfitting. However, our sample size is larger than other available studies for the study of sacroiliitis with radiomics. Manual segmentation of each joint individually may limit the ability to detect bilateral or symmetrical findings, which are significant indicators of specific conditions such as Ankylosing Spondyloarthropathy. Although the ASAS criteria do not explicitly include bilateral lesions in axial spondyloarthropathy, the modified New York criteria take this into account as they reduce the definitive radiographic sacroiliitis threshold for grade 2 lesions if observable on both sides [2], suggesting that a more comprehensive approach to joint segmentation might have provided different results.

## 5. Conclusions

This study demonstrates the potential of radiomics in enhancing the detection of active inflammatory sacroiliitis. Utilizing axial fluid-sensitive MRI images from 354 sacroiliac joints, we deployed an Extreme Gradient Boosting (XGBoost) model, which achieved an AUC-ROC of 0.71, showing promising diagnostic precision. Such a radiomics model could, following robust external validation, be incorporated in a PACS module in order to assist the diagnostic decisions of radiologists, especially those not specialized in MSK imaging. The prospective use of this algorithm is also worth exploring in future research since the algorithm has been currently tested on retrospective data. The adoption of such technologies could notably improve patient outcomes by facilitating early intervention and optimizing disease management. 

## Figures and Tables

**Table 1:** Classification models, their respective AUC-ROC scores, and 95% CI.

Classification Model	AUC-ROC	95% CI
Logistic Regression	0.61	0.47–0.75
Support Vector Machine	0.61	0.48–0.75
Random Forest	0.66	0.52–0.79
Extreme Gradient Boosting	0.71	0.58–0.84

**Table 2:** Comparative analysis of key MRI-based radiomics studies in distinguishing positive and negative cases of inflammatory sacroiliitis.

Study Details	Kepp et al. [17]	Faleiros et al. [22]	Ye et al. [37]	Current Study
No. of participants	90	56	638	177
MRI sequences	T1W-CE, STIR(T2W) *	STIR (T2W)	T2W-FS **	STIR (T2W), PD-FS ***
Positive/negative cases	33%	43%	67%	26%
Methodology	Feature-based ML	Feature-based ML	Feature-based ML	Feature-based ML
Classification models	Log Reg	SVM, k-NN, ANN	Multivariate Log Reg	Log Reg, SVM, RF, XGBoost
Optimal performance (AUC or accuracy in validation set)	0.89	0.80	0.90	0.71

* STIR: Short Tau Inversion Recovery (STIR); ** FS: fat suppressed; *** PD: proton density.

## Data Availability

The dataset will be made available by the corresponding author upon reasonable request.
